# Nontuberculous Mycobacterial Ocular Infections—Comparing the Clinical and Microbiological Characteristics between *Mycobacterium abscessus* and *Mycobacterium massiliense*

**DOI:** 10.1371/journal.pone.0116236

**Published:** 2015-01-12

**Authors:** Hsiao-Sang Chu, Shan-Chwen Chang, Elizabeth P. Shen, Fung-Rong Hu

**Affiliations:** 1 Department of Ophthalmology, National Taiwan University Hospital, Medical College, National Taiwan University, Taipei, Taiwan; 2 Division of Infectious Disease, Department of Internal Medicine, National Taiwan University Hospital, Medical College, National Taiwan University, Taipei, Taiwan; Cambridge University, UNITED KINGDOM

## Abstract

**Purpose:**

To analyze the clinical characteristics of nontuberculous mycobacterial (NTM) ocular infections and the species-specific *in vitro* antimicrobial susceptibility.

**Material and Methods:**

In 2000 to 2011 at the National Taiwan University Hospital, multilocus sequencing of *rpoB*, *hsp65* and *secA* was used to identify NTM isolates from ocular infections. The clinical presentation and treatment outcomes were retrospectively compared between species. Broth microdilution method was used to determine the minimum inhibitory concentrations of amikacin (AMK), clarithromycin (CLA), ciprofloxacin (CPF), levofloxacin (LVF), moxifloxacin (MXF) and gatifloxacin (GAF) against all strains. The activities of antimicrobial combinations were assessed by the checkerboard titration method.

**Results:**

A total of 24 NTM strains (13 *Mycobacterium abscessus* and 11 *Mycobacterium massiliense*) were isolated from 13 keratitis, 10 buckle infections, and 1 canaliculitis cases. Clinically, manifestations and outcomes caused by these two species were similar and surgical intervention was necessary for medically unresponsive NTM infection. Microbiologically, 100% of *M. abscessus* and 90.9% of *M. massiliense* ocular isolates were susceptible to amikacin but all were resistant to fluoroquinolones. Inducible clarithromycin resistance existed in 69.3% of *M. abscessus* but not in *M. massiliense* isolates. None of the AMK-CLA, AMK-MXF, AMK-GAF, CLA-MXF and CLA-GAF combinations showed synergistic or antagonistic effect against both species *in vitro*.

**Conclusions:**

*M. abscessus* and *M. massiliense* are the most commonly identified species for NTM ocular infections in Taiwan. Both species were resistant to fluoroquinolones, susceptible to amikacin, and differ in clarithromycin resistance. Combined antimicrobial treatments showed no interaction *in vitro* but could be considered in combination with surgical interventions for eradication of this devastating ocular infection.

## Introduction

Nontuberculous mycobacteria (NTM), also known as atypical mycobacteria, are opportunistic pathogens that live in natural environments including water, soil, food, and air.[1] In the past twenty years, the number of NTM ocular infections has gradually increased and the majority of the infections was caused by the rapidly growing mycobacteria (RGM).[2,3] Penetrating ocular trauma or surgery can introduce NTM from the environment into ocular tissues.[4] The coexistence of foreign body or biomaterial, exposure to contaminated water, and local or systemic immunosuppression of host are other risk factors of NTM eye infections.[5,6] After the1990s’ with the growing popularity of laser refractive surgery or keratoplasty, NTM keratitis has been more widely recognized and thus commonly reported.[3,7] Other sporadic NTM ocular infections include buckle infections, conjunctivitis, canaliculitis, scleritis, choroiditis, endophthalmitis and external adnexal infections.[8]

Traditionally, NTM were classified by use of a combination of laboratory techniques, such as growth rate, smear morphology, biochemical characteristics and antimicrobial profiles.[9,10] Among the RGM, *Mycobacterium abscessus, M. chelonae* and *M. fortuitum* groups were reported most commonly for ocular infections.[8] Recently, with the advent of molecular identification using gene sequencing, nomenclature of NTM has changed and identified new subgroups. Between 2004 and 2006, the species *M. abscessus* (also called *M. abscessus* sensu lato) was further subclassified into three species, *M. abscessus* (*M. abscessus* sensu stricto), *M. massiliense*, and *M. bolletii*.[11] Although the availability of gene sequencing has had tremendous influence on the taxonomy of mycobacteria, there has not been any studies comparing the drug response among different *M. abscessus* species in ocular disease. Therefore, our study had two main purposes. The first is to investigate the heterogeneity of NTM ocular isolates by multilocus gene sequencing and to compare their clinical presentations, treatments, and outcomes. The second is to evaluate the *in vitro* species-specific drug susceptibility to single and combined antimicrobial agents. We hope that by categorizing NTM into species level, more information for treating this potentially devastating ocular infection may be deduced.

## Material and Methods

### Clinical Data

We conducted this study in adherence to the Declaration of Helsinki. Our study had two parts, the clinical part of participants’ treatment outcomes and the microbiological part of drug susceptibility. The clinical part of this study was approved by the Institutional Review Board of National Taiwan University Hospital (NTUH) (IRB No.:201203034RIB) with waiver of documentation of consent due to that it was a retrospective chart review with adequate encryption of participants’ personal data. Our IRB committee also approved the microbiological part of this study. Human Biobank Management Act was not applicable to the clinical isolates in this study. A retrospective chart review was performed for patients with ocular infection that had a positive isolation of NTM by the mycobacteriology laboratory of Department of Laboratory Medicine, NTUH between January 2000 and December 2011. Data collected included patient demographics, systemic and local risk factors, source of positive cultures, clinical courses, local findings and treatment outcomes.

### Bacterial strains

Ocular samples were collected by a swab with all-in-one collection-transport system (Culturette, Becton Dickinson and Co., Cockeysville, Maryland, USA) and sent to the central microbiological laboratory to be spread onto Lowenstein-Jensen (L-J) slants and tested by the fluorometric BACTEC system (BACTEC *Mycobacterium* Growth Indicator tube 960 System; Becton, Dickinson and Company). NTM were initially identified by conventional biochemical methods. NTM isolates were then stored in trypticase soy broth containing 5% glycerol at −70°C and on L-J slants for further molecular identification.

### DNA isolation, polymerase chain reactions (PCRs), and sequencing

DNA was extracted from a 10μL loopful of each mycobacterial colony by use of a Blood & Tissue Genomic DNA Extraction Miniprep System (VIOGENE, New Taipei City, Taiwan, R.O.C.) according to the manufacturer’s instructions. PCRs were carried out with TaKaRa Taq (TAKARA BIO Inc, Shiga, Japan). Partial amplification of the ***hsp65*** gene was performed with primers Tb11 (5’-ACC AAC GAT GGT GTG TCC AT-3’) and Tb12 (5’-CTT GTC GAA CCG CAT ACC CT-3’). A 401-bp sequence was derived from the amplicon by using the same primer pair. Partial amplification of the ***rpoB*** sequence was carried out with the primer pair Myco-F (5’-GGC AAG GTC ACC CCG AAG GG-3’) and Myco-R (5’-AGC GGC TGC TGG GTG ATC ATC-3’). A 711-bp sequence was derived from the amplicon by using the same primer pair. Partial amplification of the ***secA*** gene was carried out with primers Mtu.For1 (5’-GAC AGY GAG TGG ATG GGY CGS GTG CAC CG-3’) and Mtu.Rev490 (5’-GCG GAC GAT GTA RTC CTT GTC SCG-3’) and a 465-bp sequence were derived from the amplicon by using the same primer pair. The sequencing results were then input to the bioinformatics software, BioNumerics 6.0 (Applied Maths, Sint-Martens-Latem, Belgium) and compared with the database. Species identification was considered unambiguous only when there was agreement in the species assignation by *hsp65, rpoB* and *secA* gene sequencing analysis.[12]

### Broth microdilution

Serial double dilutions of standard powder of amikacin (AMK), clarithromycin (CLA), ciprofloxacin (CPF), levofloxacin (LVF), moxifloxacin (MXF) and gatifloxacin (GAF) (Sigma-Aldrich Co., St. Louis, Missouri) were prepared at a concentration range of 0.06 to 32μg/ml according to Clinical and Laboratory Standards Institute (CLSI) recommendations.[13] The bacteria were inoculated into cation-adjusted Muller-Hinton (M-H) broth to reach a final concentration of 10^5^ colony forming units (CFU) /ml. The inoculated microdilution trays were sealed in plastic bags and incubated at 30°C in ambient air. The minimum inhibitory concentration (MIC) was defined as the lowest concentration of drug that inhibited visible growth. Except clarithromycin, all trays were examined at Day 3. In order to monitor whether there was any change in MIC, we examined the susceptibility results again at Day 5. The reading for clarithromycin was performed after 3, 5 and 14 days of incubation for detection of inducible resistance. The MICs of *Staphylococcus aureus* ATCC 29213 and *Pseudomonas aeruginosa* ATCC 27853 after 24 hours of incubation, and *M. fortuitum* ATCC 6841 after 72 hours of incubation were determined to serve as internal controls.

### Checkerboard titration methods

Checkerboard titration method was used to assess the effect of combining two antimicrobial agents against each strain. The activity of drug combinations was tested for amikacin-clarithromycin (AMK-CLA), amikacin-moxifloxacin (AMK-MXF), amikacin-gatifloxacin (AMK-GAF), clarithromycin-moxifloxacin (CLA-MXF), and clarithromycin-gatifloxacin (CLA-GAF). Serial dilutions of two different antimicrobial agents were mixed in cation-adjusted M-H broth using microdilution plates. Bacteria were inoculated to reach a final concentration of 10^5^ CFU/ml in each well. In order to detect inducible drug resistance, all plates were sealed in plastic bags and incubated at 30°C for 14 days before MIC determination. The fractional inhibitory concentration (FIC) for each combination which inhibited growth was calculated based on the following formula: FIC = MIC of drug A in combination /MIC of drug A alone + MIC of drug B in combination/MIC of drug B alone. Then the minimum FIC of the calculated FICs was defined as the FIC index. Synergism was defined as a FIC index of <0.5, and antagonism was defined as a FIC index of >4. A FIC index between 0.5 and 4 was regarded as no interaction.[14] The MICs of *Staphylococcus aureus* ATCC 29213 and *Pseudomonas aeruginosa* ATCC 27853 after 24 hours of incubation, and *M. fortuitum* ATCC 6841 after 72 hours of incubation were all used as the internal controls.

### Statistics

Statistical analyses were performed by using SPSS 17.0 (IBM Corporation, USA). The results were expressed as ranges and means or as numbers of patients. Continuous variables were analyzed by the non-parametric Mann-Whitney U test. All *P* values were two sided, and a *P* value of <0.05 indicated statistical significance.

## Results

### Identification of Clinical Isolates

Our study recruited 39 patients with culture-proven NTM ocular infections between January 2000 and December 2011 (Table 1). Among them, twenty-four clinical isolates were preserved for molecular identification, and for patients with more than one stored isolate, the earliest isolate was selected. These isolates were collected from patients of keratitis (*n* = 13), scleral buckle infection (*n* = 10), and canaliculitis (*n* = 1). The other 15 isolates collected in the early study period were identified by conventional biochemical methods only and not included for microbiological study due to loss of viability or indistinguishable labeling. These 15 lost isolates were collected from patients of keratitis (n = 10), buckle infection (n = 4), and canaliculitis (n = 1).

**Table 1:** Conventional Biochemical Identification vs. Molecular Identification.

**Results of Conventional Biochemical Identification (Total number = 39)**	**Isolates Available for Molecular Identification (Total number = 24)**	**Results of Molecular Identification (Total number = 24)**
*M. abscessus* group (26)	*M. abscessus* group (19)	*M. abscessus* (10) *M. massiliense* (9)
*M. chelonae* group (5)	*M. chelonae* group (2)	*M. massiliense* (2)
*M. fortuitum* group (4)	*M. fortuitum* group (1)	*M. abscessus* (1)
*M. avium* group (2)	*M. avium* group (0)	*M. avium* group (0)
Mycobacteria spp.* (2)	Mycobacteria spp.* (2)	*M. abscessus* (2)

*Mycobacteria spp.: mycobacteria species that failed to be further identified by conventional biochemical methods.

Among the 13 isolates from keratitis patients, six were *M. abscessus* and 7 were *M. massiliense*. Among the 10 isolates from buckle infection patients, six were *M. abscessus* while 4 were *M. massiliense*. And the isolate from canaliculitis patient was identified as *M. abscessus.* We compared the conventional biochemical identifications and those obtained from molecular method (Table 1). Ten of the 19 isolates that were initially identified as *M. abscessus* group were re-classified as *M. abscessus* and the remaining 9 were *M. massiliense.* The two isolates conventionally identified as *M. chelonae* group were re-identified as *M. massiliense*. The one isolate conventionally identified as *M. fortuitum* group was re-classified as *M. abscessus*. The two mycobacteria that initially failed to be identified to the species level by conventional methods were reported as *M. abscessus*. To sum up, according to the multilocus gene sequencing, a total of 13 *M. abscessus* and 11 *M. massiliense* were included for clinical and microbiological comparison.

### Baseline Characteristics and Clinical Features

As Table 2 shows, patients with *M. abscessus* (n = 6) and *M. massiliense* (n = 7) keratitis shared similar demographic characteristics. No significant differences were noted in the clinical presentation and the initial or final visual outcomes between the two groups. Risk factors related to NTM keratitis were often related to trauma or operation. The diagnoses were often delayed and the treatments were usually prolonged in both groups. Although antimicrobial treatment could eradicate 33.3% (2 out of 6) *M. abscessus* keratitis and 42.9% (3 out of 7) *M. massiliense* keratitis, combined surgical managements were necessary to treat medically unresponsive *M. abscessus* and *M. massiliense* keratitis.

**Table 2:** Demographic Characteristics and Clinical Manifestations of NTM Keratitis (n = 13).

	***M. abscessus* (n = 6)**	***M. massiliense* (n = 7)**	***P value* (Mann-Whitney U test)**
**Age**	49 to 81(63.5 ± 26.8)	12 to 86 (49.3 ± 21.9)	**0.568**
**F:M**	2:4	3:4	
**Risk Factors**			
Trauma / Operation	1/6	3/7	
Local/ Systemic Immunosuppression	1/6	4/7	
**Initial Presentation**			
Epithelial defect	6/6	4/7	
Hypopyon	1/6	1/7	
Corneal Melting	3/6	0/7	
**Initial BCVA**			
Worse than 20/400	6/6	5/7	
20/400 to 20/40	0/6	2/7	
Better than 20/40	0/6	0/7	
**Time form onset to diagnosis** **(mean ± SD)**	3 to 35 days (18.3 ± 14.7 days)	3 to 180 days (60.7 ± 61.1 days)	**0.173**
**Antimicrobial Treatment Only**	2/6	3/7	
monotherapy: combincation therapy	0:2	1:2	
**Combined Surgical Treatment**	4/6	4/7	
**Time for Resolution**	30 to 240 days (180 ± 83.2 days)	14 to 120 days (57.3 ± 36.3 days)	**0.064**
**Final BCVA**			
Worse than 20/400	3/6	5/7	
20/400 to 20/40	2/6	1/7	
Better than 20/40	1/6	1/7	

Patients with *M. abscessus* (n = 6)and *M. massiliense* (n = 4) buckle infections also shared similar baseline characteristics. The mean age was 62.2 ± 14.2 and 55.5 ± 19.5 in *M. abscessus* and *M. massiliense* group (*p* = 0.55). Previous buckle surgery was the only remarkable risk factor in both groups. Buckle infections cases due to *M. abscessus* or *M. massiliense* shared indistinguishable clinical features such as conjunctival injection and purulent discharge. One patient of *M. abscessus* buckle infection progressed to endophthalmitis and resulted in enucleation of his painful blind eye. All other 9 buckle infections caused by both species resolved after surgical removal of buckle material. Topical antimicrobials such as gentamicin and levofloxacin were empirically used for 1 to 8 weeks after buckle removal. The time needed from buckle removal to complete disease resolution was 28.0 ± 23.3 (between 8 and 60) days in *M. abscessus* and 33.3 ± 19.3 (between 9 and 54) days in *M. massiliense* groups (*p* = 0.74).

The one canaliculitis case caused by *M. abscessus* resolved after nasolacrimal duct irrigation with gentamicin/amikacin twice combined with topical levofloxacin use for 5 months.

### Drug Susceptibility Tests of Single Antimicrobial Agents

Table 3 shows the *in vitro* susceptibility results for 13 isolates of *M. abscessus* and 11 isolates of *M. massiliense* that were tested by broth microdilution method. The results show that 100% of *M. abscessus* isolates and 90.9% of *M. massiliense* isolates were susceptible or intermediate susceptible to amikacin. The minimum concentration required inhibiting the growth of 90% (MIC_90_) of *M. abscessus* and *M. massiliense* isolates were 16μg/ml and 32μg/ml. All *M. abscessus* and *M. massiliense* isolates were resistant to fluoroquinolones with MIC ranging from 4 to more than 32μg/ml after incubation for 5 days. Few strains showed intermediate susceptible to fluoroquinolones on Day 3 were confirmed to be resistant on Day 5.

**Table 3:** Susceptibility Interpretation of Broth Microdilution Test.

**NTM species**	***M. abscessus***						***M. massiliense***					
**Antibiotics**	**Day 3**			**Day 5**			**Day 3**			**Day 5**		
	MIC*range	**%SI^※^**	**MIC_90_^#^**	**MIC range**	**%SI**	**MIC_90_**	**MIC range**	**%SI**	**MIC_90_**	**MIC range**	**%SI**	**MIC_90_**
**Amikacin (AMK)**	8 to 16	100	16	8 to 16	100	16	8 to >32	90.9	32	8 to >32	90.9	32
**Ciprofloxacin (CPF)**	4 to 32	0	32	8 to >32	0	>32	1 to >32	27.3	8	4 to >32	0	32
**Levofloxacin (LVF)**	16 to 32	0	32	32 to >32	0	>32	8 to 32	0	32	16 to >32	0	>32
**Moxifloxacin (MXF)**	4 to 16	0	16	8 to 32	0	32	1 to 16	9.1	16	4 to >32	0	32
**Gatifloxacin (GAF)**	1 to 16	30.7	8	8 to 32	0	32	1 to 16	18.2	8	16 to 32	0	32

Antimicrobial MIC break points (S,I,R): AMK(16,32,64); CPF(1,2,4); LVF(1,2,4); MXF(1,2,4); GAF(1,2,4) (μg/mL) (S, I, R: susceptible, intermediate susceptible and resistant)

*MIC(μg/mL): minimum inhibitory concentration

^※^%SI: percentage of susceptible or intermediate susceptible to antibiotics

^#^MIC_90_: MIC required to inhibit the growth of 90% of the organism

### Extended Screening on Inducible Resistance to Clarithromycin

The emergence of resistance may occur in *M. abscessus* or *M. massiliense* after prolonged incubation of more than 14 days with clarithromycin. Table 4 shows the susceptibility of *M. abscessus* and *M. massiliense* to clarithromycin recorded at different time points. After 14 days of prolonged incubation, 69.3% of *M. abscessus* isolates turned to be resistant to clarithromycin with MIC_90_ exceeded 32μg/ml. However, none of *M. massiliense* isolates showed resistance to clarithromycin with MIC_90_ equal to 0.125μg/ml after 14 days of incubation.

**Table 4:** Extended Screening on Inducible Resistance to Clarithromycin (CLA).

**NTM species**		**Day 3**	**Day 5**	**Day 14**
***M. abscessus***	MIC* range	≤ 0.06 to 1	≤ 0.06 to 4	≤ 0.06 to > 32
	MIC_90_^#^	0.5	4	> 32
	%R^※^	0	0	69.3
***M. massiliense***	MIC range	≤ 0.06	≤ 0.06 to 0.125	≤ 0.06 to 0.125
	MIC_90_	≤ 0.06	0.125	0.125
	%R	0	0	0

CLA break points (S, I, R):(2,4,8) (μg/mL) (S, I, R: susceptible, intermediate susceptible and resistant)

*MIC(μg/mL): minimum inhibitory concentration

^※^%R: percentage of resistance to antibiotics

^#^MIC_90_: MIC required to inhibit the growth of 90% of the organism

### Drug Susceptibility Tests of Combined Antimicrobial Agents

As Table 5 shows, no antimicrobial combination showed synergism (FIC index < 0.5) or antagonism (FIC index > 4). All combinations had no interactions against 13 *M. abscessus* and 11 *M. massiliense* isolates with FIC index between 0.5 and 4.

**Table 5:** Susceptibility Tests of Combined Antimicrobial Agents.

**Species and Agents**	**Mean FIC index ± SD**	**Median FIC index**	**% of isolates with combination activity**		
			**Synergism**	**No Interaction**	**Antagonism**
***M. abscessus (n = 13)***					
**AMK-CLA**	2.00 ± 0.00	2	0	100	0
**AMK-MXF**	1.11 ± 0.65	1	0	100	0
**AMK-GAF**	1.58 ± 0.49	2	0	100	0
**CLA-MXF**	1.56 ± 0.74	2	0	100	0
**CLA-GAF**	1.01 ± 0.47	0.75	0	100	0
***M. massiliense (n = 11)***					
**AMK-CLA**	1.82 ± 0.25	2	0	100	0
**AMK-MXF**	1.38 ± 0.61	1	0	100	0
**AMK-GAF**	1.84 ± 0.59	2	0	100	0
**CLA-MXF**	1.96 ± 0.56	2	0	100	0
**CLA-GAF**	2.00 ± 0.00	2	0	100	0

### Analysis of in vitro Antimicrobial Susceptibility Results with Clinical Management of NTM Keratitis Cases

Table 6 summarizes the *in vitro* antimicrobial susceptibility results and clinical management of the 13 NTM keratitis patients. For NTM keratitis, we gave empirical treatments first and adjusted medication according to the susceptibility reports and clinical responses. Topical amikacin was used empirically for 11 cases. Topical or systemic fluoroquinolones were also used for 9 cases. Clarithromycin eye drops were not easily available in Taiwan, therefore, only 3 patients received clarithromycin treatment as part of the combination therapy. Five cases, two *M. abscessus* and 3 *M. massiliense* keratitis, reached complete resolution without surgical intervention. The other 8 cases required surgical intervention.

**Table 6:** Antimicrobial Susceptibility Results & Clinical Management of NTM Keratitis Cases.

**NTM species**	**No.**	**Antimicrobial Susceptibility**			**Medication Given**			**Surgical Treatments**
		**CLA**	**AMK**	MFX*	**CLA**	**AMK**	FQ**	
*M. abscessus*	1	R	S	R	−	+	+	−
	2	R	S	R	−	+	+	−
	3	R	S	R	−	+	−	LK^⧺^ + therapeutic PKP^⧺⧺^
	4	R	S	R	−	−	+	Enucleation
	5	R	S	R	−	+	−	Therapeutic PKP
	6	R	S	R	−	+	+	LK + therapeutic PKP
*M. massiliense*	7	S	S	R	−	+	−	−
	8	S	S	R	−	+	+	−
	9	S	S	R	−	+	+	−
	10	S	S	R	+	+	−	Flap amputation
	11	S	S	R	−	−	+	Flap lifting and irrigation
	12	S	S	R	+	+	+	LK + vitrectomy
	13	S	S	R	+	+	+	LK

S/R: susceptible/resistant; +: medication has been given; −: medication has not been used

*MXF: since *M. abscessus/massiliense* have similar susceptibility patterns to all generation of fluoroquinolones, here we use moxifloxacin as representative fluoroquinolones

**FQ: means any kind of fluoroquinolones was used in the treatment course

^⧺^LK: lamellar keratectomy

^⧺⧺^PKP: penetrating keratoplasty

## Discussion

To the best of our knowledge, our paper is first to focus on the clinical relevance and microbiological response between *M. abscessus* and *M. massiliense* ocular infections. Our results showed that after the year of 2000, *M. abscessus* and *M. massiliense* were the most commonly identified NTM ocular pathogens at our hospital. The baseline characteristics, initial presentations and final outcomes of patients with *M. abscessus* and *M. massiliense* ocular infections were similar. Moreover, most NTM keratitis required not only surgical treatment but also prolonged antimicrobial use. In contrast, NTM buckle infections may quickly resolve after removal of the buckle material. The *in vitro* susceptibility results showed that ocular isolates of *M. abscessus* and *M. massiliense* were generally susceptible to amikacin but resistant to fluoroquinolones. *M. abscessus* behaved differently from *M. massiliense* in the inducible macrolide resistance. In addition, combination of two antimicrobial agents showed no interaction against *M. abscessus* and *M. massiliense in vitro.*

For the past decade, NTM associated disease incidence increased from 2.7 to 10.2 cases per 100,000 patients in Taiwan. The most frequent clinical isolates were *M. avium* group (30.0%), followed by *M. abscessus* group (17.5%), *M. fortuitum* group (13.0%), and *M. chelonae* group (9.6%). [15] During the study period, our central microbiological laboratory received a total of 14909 *M. tuberculosis* clinical isolates and 13730 NTM isolates. Among them, there was only one tuberculosis ocular isolate and 39 NTM ocular isolates. Thus, NTM was concluded to be much common than tuberculosis in external ocular infections. We further found that the *M. abscessus* group, including *M. abscessus* and *M. massiliense*, was the most common pathogen of ocular infection. The rapidly growing mycobacteria identified by conventional methods before were actually re-identified as *M. abscessus* group by molecular methods. Comparing with our previous report of NTM keratitis during the 1990’s,[16] we have found that the frequency of *M. abscessus* group clinical isolates increased dramatically while the frequency of *M. chelonae/fortuitum* groups decreased. We speculate that this change could possibly be related to the extensive use of fluoroquinolones in the ophthalmic field. Fluoroquinolones were proven to be more active against *M. fortuitum* and *M. chelonae* groups.[10,17] Therefore, the quinolone-resistant strains, like *M. abscessus* group, could be selected and led to opportunistic infections. In addition, conventional methods of species identification may be less accurate due to the low reproducibility of biochemical tests, ambiguous phenotyping, and limited database of phenotypic characteristics of different strains.[18] In fact, the so-called *M. chelonae* and *M. fortuitum* groups that were observed in earlier studies could very possibly be *M. abscessus* group. We also observed the misclassification by conventional biochemical identification in our study (Table 1).

The recent development of molecular identification has greatly changed the taxonomy of NTM.[19] 16S rRNA gene sequencing method has been used for the identification of RGM in few ophthalmology studies.[17] However, since *M. abscessus, M. massiliense,* and *M. bolletii* share nearly 100% sequence similarity of 16S rRNA gene, differentiation is best achieved by analysis of polymorphism in other housekeeping genes that are highly conservative in all NTM species, for example, the *rpoB, hsp65, sodA, secA* gene, or the 16S-23S rRNA internal transcribed sequences.[20] In our study, *rpoB, hsp65*, and *secA* were chosen for multilocus gene sequencing to further differentiate *the M. abscessus* group.[12] *M. massiliense* was then found to account for 45.8% (11 out of 24) of our originally identified ocular *M. abscessus* group. The proportion of *M. massiliense* strains among *M. abscessus* group may vary by different geographic location. *M. massiliense* accounted for 33.3% of all clinical isolated *M. abscessus* group in one cohort study in the U.S.[12] Presently, there is no explanation for the variance in different geographic areas. The percentage of NTM ocular infections by these two species may also vary by different disease entity.

Medical treatment of NTM ocular infections has always been challenging. Aminoglycosides, macrolides, and fluoroquinolones have been proposed to be the drugs of choice in treatment of NTM.[8,10] In a recent retrospective study of ocular NTM infections, Brown-Elliott *et al* reported that the percentage of their isolates in the *M. abscessus* group that were susceptible/intermediate susceptible (SI) to amikacin, clarithromycin, ciprofloxacin, levofloxacin, moxifloxacin and gatifloxacin were 100%, 100%, 21.6%, 5.4%, 21.6% and 48.6%, respectively.[17] When compared with the aforementioned results, our isolates showed a similarly high percentage of susceptibility to amikacin and low sensitivity to all fluoroquinolones. The aforementioned paper did not mention the possible inducible clarithromycin resistance, while we found that a high proportion (69.3%) of *M. abscessus* isolates with inducible clarithromycin resistance. Clarithromycin is a bacteriostatic macrolide that inhibits bacterial protein synthesis by binding of 23S rRNA. The macrolide resistance among mycobacteria is related to erythromycin ribosome methyltransferase *(erm)* gene. *Erm* gene encodes methyltransferase that causes methylation of 23S rRNA and therefore interferes the binding of macrolides to its subunit. Comparing with the sequence of *erm*(41) of *M. abscessus*, the *erm*(41) of *M. massiliense* has a critical deletion that translates to a dysfunctional methyltransferase that poorly binds to 23S rRNA with or without macrolide exposure. Therefore, inducible macrolide resistance usually will not be observed in most *M. massiliense* unless a point mutation on 23S rRNA occurs that reverses the methyltransferase-23rRNA binding activity. [21,22] The observation of inducible clarithromycin resistance of *M. abscessus* has not yet been reported by any other study devoted to NTM ocular infections. Thus, although clarithromycin may be an effective NTM treatment, prolonged use may actually induce resistance and eventually result in uncontrollable infection.

Many *in vitro* studies uniformly suggest that amikacin, a bactericidal aminoglycoside that inhibits bacterial protein synthesis by binding of bacterial 16S rRNA, is the drug of choice for the treatment of RGM.[2,17] Our *in vitro* results confirmed that amikacin was highly effective against our ocular isolates of *M. abscessus* (100%) and *M. massiliense* (90.9%). However, there was only one *M. massiliense* keratitis cured by amikacin monotherapy in our 13 keratitis patients (Table 6). Failure of treatment with amikacin alone has also been reported due to its poor penetration through corneal epithelium.[6, 23] Fluoroquinolones are bactericidal antibiotics that inhibit DNA gyrase A or DNA topoisomerase IV. *M. abscessus* and *M. massiliense* have been reported with variable or poor sensitivity to fluoroquinolones.[2,10,17] In our study, 100% of the isolates were resistant to all fluoroquinolones *in vitro*. Clinically, fluoroquinolones were used in combination with amikacin in 9 of our 13 microbial keratitis patients. Only 4 patients responded to the combination therapy, while the others needed surgical interventions (Table 6).

Through our clinical outcome analysis, we discovered that the management of NTM buckle infection and corneal infection were different. Our study showed that surgical removal of buckle material was the key step for the eradication of NTM buckle infections. Surgical removal of the foreign body also prevented the possibility of biofilm formation. It is important to mention that the use of topical antibiotics for NTM buckle infections played a minor role in the treatment course since retrospective review of these patients found that the topical antibiotics used were not effective against NTM. In contrast to buckle infections, the treatment of NTM keratitis was much more difficult. Drug penetration through corneal epithelium and stroma are usually poor.[24] Even after surgical debulking of infiltrated cornea, prolonged use of multiple antimicrobials was often needed for the eradication of NTM keratitis.

A major limitation of our study was that it was retrospective in nature. The 24 patients enrolled in this study presented with variable disease severity and were treated by different clinicians. Thus, both the baseline clinical presentation and the managements could not be standardized for adequate comparisons. Lastly, through the duration of the study, 15 isolates out of a total 39 cases were not preserved due to various reasons. This may have possibly affected our final analyses of clinical outcomes and drug susceptibility. Nonetheless, considering the scarcity of NTM ocular infections, the present study collecting NTM strains from over 10 years may be one of the largest NTM series collected from the eye.

In conclusion, we found the *M. abscessus* group has become the most frequently isolated pathogen of NTM ocular infections with nearly equal distribution of the sub-species *M. abscessus* and *M. massiliense*. The clinical manifestations of *M. abscessus* and *M. massiliense* ocular infections were similar and early surgical treatments were often needed to control the infection. Microbiologically, we noticed an increase in fluoroquinolone resistance. Also, our *in vitro* results showed that amikacin remained the most effective antibiotic for both *M. abscessus* and *M. massiliense.* The novel finding that prolonged incubation with clarithromycin may induce drug resistance in *M. abscessus* should alarm clinicians of possible delayed poor clinical response that may require a change in therapy. Since no antagonistic effect was noted, clinically, we still recommend combined antimicrobial treatment to decrease possible drug resistance during monotherapy. However, the best antimicrobial regimen for treating NTM ocular infections needs further large-scaled, prospective studies.
